# Microbial lactate utilisation and the stability of the gut microbiome

**DOI:** 10.1017/gmb.2022.3

**Published:** 2022-05-04

**Authors:** Petra Louis, Sylvia Helen Duncan, Paul Owen Sheridan, Alan William Walker, Harry James Flint

**Affiliations:** 1 Gut Health Group, Rowett Institute, University of Aberdeen, Aberdeen, UK; 2 School of Biological Sciences, University of Aberdeen, Aberdeen, UK

**Keywords:** gut microbiota, anaerobic fermentation, lactate, shortchain fatty acids, cross-feeding

## Abstract

The human large intestinal microbiota thrives on dietary carbohydrates that are converted to a range of fermentation products. Short-chain fatty acids (acetate, propionate and butyrate) are the dominant fermentation acids that accumulate to high concentrations in the colon and they have health-promoting effects on the host. Although many gut microbes can also produce lactate, it usually does not accumulate in the healthy gut lumen. This appears largely to be due to the presence of a relatively small number of gut microbes that can utilise lactate and convert it to propionate, butyrate or acetate. There is increasing evidence that these microbes play important roles in maintaining a healthy gut environment. In this review, we will provide an overview of the different microbes involved in lactate metabolism within the gut microbiota, including biochemical pathways utilised and their underlying energetics, as well as regulation of the corresponding genes. We will further discuss the potential consequences of perturbation of the microbiota leading to lactate accumulation in the gut and associated disease states and how lactate-utilising bacteria may be employed to treat such diseases.

## Introduction

Lactate is produced by many different anaerobic bacteria from the fermentation of energy sources, especially carbohydrates. For some gut bacteria, such as lactobacilli, streptococci and bifidobacteria, it is a major fermentation product, but many other species are known to form lactate in pure culture as a minor, or alternative, product depending on the growth conditions. Despite this, colonic concentrations of lactate in adults are normally low relative to those of the major short-chain fatty acids produced by microbiota fermentation (acetate, propionate and butyrate). This reflects in part the relative abundance of lactate-producing species, but also the regulation of fermentative pathways and metabolic cross-feeding, especially the utilisation of lactate by a subset of lactate-utilising intestinal bacteria (Belenguer et al., 2006; Counotte et al., 1983; Duncan et al., 2004).

**Figure 1:**
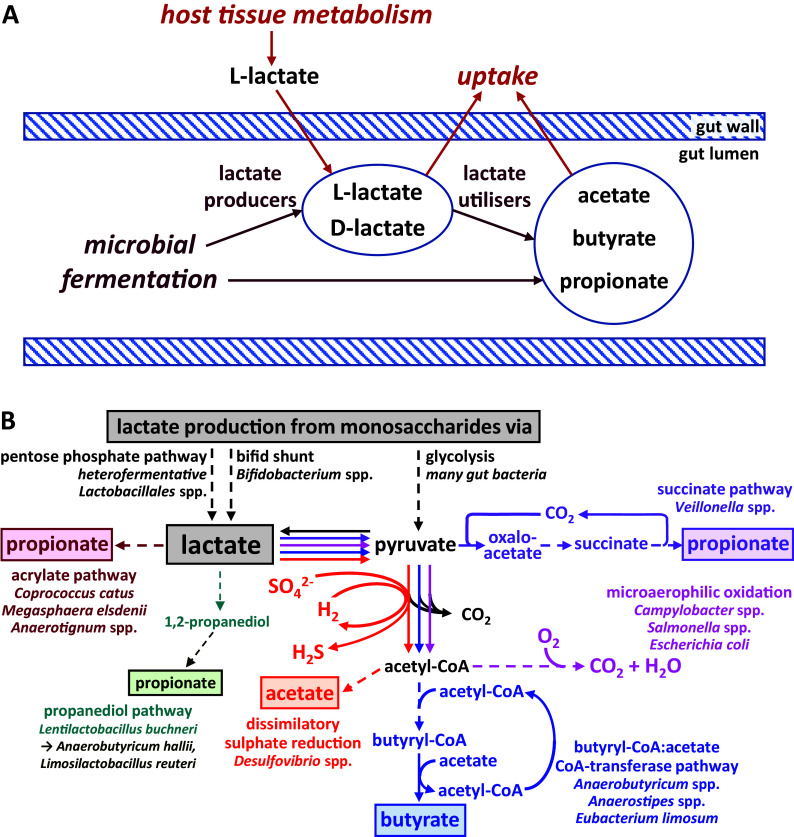
Lactate metabolism in the intestine. (A) Overview of lactate production and utilisation by host and microbiota and transport across the gut epithelium. (B) Biochemical pathways employed by different gut microbes for the production and utilisation of lactate. Pathway sections with multiple enzymatic steps are shown as dashed lines. Only the general flow from substrates to key intermediates and products is shown; exact stoichiometries are not included.

The phenomenon of lactic acidosis has been known for many years as an important issue in animal nutrition. In ruminants, this state can be triggered by diets high in readily fermentable carbohydrates. This can result in the rapid promotion of lactate-producing bacteria within the rumen microbiota leading to a drop in rumen pH, which decreases the growth of the normally dominant rumen anaerobic microbiota and further promotes low pH-tolerant lactic acid-producing species. Unless it can be reversed, the condition can prove fatal as the D-isomer of lactate is a neurotoxin (Chan et al., 1994). Lactate metabolism also plays an important role in the microbial ecology of pathogens and in the physiology of host tissues at the gut mucosa, with the host providing an additional source of L-lactate entering the gut (Figure 1A).

This article will provide an overview of microbial lactate metabolism in the microbial communities of the human intestine, including the biochemical pathways employed by gut bacteria involved in formation and utilisation of lactate as well as microbial community interactions. We will consider the role of lactate in destabilising the ecosystem of the large intestine in the light of recent work on differential growth inhibition of dominant bacterial groups by lactate. Furthermore, the key role of lactate-utilising bacteria and lactate-inducible gene clusters in maintaining ecosystem stability and preventing lactate-induced perturbations (acidosis) will be covered. Finally, we will highlight the potential of lactate-utilising bacteria as live biotherapeutic products.

## Biochemistry and energetics of lactate metabolism

### Biochemical pathways and corresponding microbes involved in lactate production and utilisation

Lactate production is widespread among human gut microbes, whereas its utilisation appears to be restricted to comparatively fewer species and genera (Figure 1B). The predominant pathway for lactate production from most monosaccharides (originating from dietary fibre breakdown) is glycolysis and subsequent reduction of the resulting pyruvate. It is carried out by many different gut bacteria belonging to the dominant phyla of Bacteroidetes, Firmicutes (including homofermentative *Lactobacillus* and *Streptococcus* species within the order Lactobacillales, which generate principally lactate as an acidic fermentation end product) and Proteobacteria (Gottschalk, 1986). Furthermore, heterofermentative Lactobacillales species produce lactate as one of several products via the pentose phosphate pathway (Salvetti et al., 2013). *Bifidobacterium* species within the phylum Actinobacteria, and possibly also bacteria within the Coriobacteriales order of Actinobacteria, utilise a different pathway for lactate production, the bifid shunt (Gupta et al., 2017). Lactate is often produced as one of several products by anaerobic bacteria to achieve overall redox balance (covered in more detail in the next section), and the relative amount of different fermentation products can vary with prevailing environmental conditions, such as pH or availability of carbon dioxide (Louis and Flint, 2017).

Lactate utilisation (Figure 1B) is carried out under microaerophilic conditions by Proteobacteria, including pathogens such as *Campylobacter* and *Salmonella* species, which fully oxidise lactate to carbon dioxide and water (Gillis et al., 2019). *Desulfovibrio* species, on the other hand, can convert lactate to acetate, together with the dissimilatory reduction of sulphate to sulphide (Marquet et al., 2009; Tang et al., 2021). Within the Firmicutes phylum, certain microbes are able to convert lactate to propionate or butyrate (Louis and Flint, 2017; Table 1). For example, *Anaerobutyricum* and *Anaerostipes* species (both *Lachnospiraceae*) produce butyrate from lactate and acetate (Duncan et al., 2004), whereas the acetogenic bacterium *Eubacterium limosum* (*Eubacteriaceae*) converts lactate to butyrate with net production of acetate (Pham et al., 2017). Several alternative pathways exist for propionate formation from lactate within the gut microbiota. The acrylate pathway has only been identified in a handful of species, including *Coprococcus catus* (*Lachnospiraceae*) and *Megasphaera elsdenii* (*Veillonellaceae*), whereas the succinate pathway is employed by *Veillonella* species, also within the *Veillonellaceae* family (Reichardt et al., 2014). Finally, propionate may also be formed from lactate via the intermediate 1,2-propanediol, but this likely involves more than one microbe. The conversion of lactate to 1,2-propanediol has been demonstrated in *Lentilactobacillus buchneri* isolated from maize silage, but closely related microbes may also be present in the human gut. Other bacteria, including *Anaerobutyricum hallii* and *Limosilactobacillus reuteri*, can convert 1,2-propanediol into propionate or propanol (Louis and Flint, 2017). Whether this route of lactate consumption makes a significant contribution to lactate metabolism in the human gut remains to be elucidated. It should be noted that many lactate utilisers can also grow on, and may prefer, other substrates (eg. Duncan et al., 2004; Reichardt et al., 2014) and re-uptake of lactate produced from inositol by *Anaerostipes caccae* for further conversion to butyrate has been demonstrated in pure culture (Bui et al., 2021).

**Table 1.** Abundant obligately anaerobic Firmicutes bacteria from the human colon shown to utilise lactate *in vitro.*

Species	Type strain^a^	Prevalence in human metagenomes^b^	Pathway^c^	Reference(s)
*Anaerobutyricum hallii (*formerly *Eubacterium hallii)*	DSM 3353^T^	77% (*n* = 62,700)	D-iLDH, butCoAT	Duncan et al., 2004; Shetty et al., 2018
*Anaerobutyricum soehngenii (*formerly *Eubacterium hallii)*	DSM 17630^T^ (=L2-7)	77% (*n* = 62,700)	D-iLDH, butCoAT	Duncan et al., 2004; Shetty et al., 2018
*Anaerostipes caccae*	DSM 14662^T^ (=L1-92)	66% (*n* = 35,300)	D-iLDH, butCoAT	Duncan et al., 2004; Schwiertz et al., 2002
*Anaerostipes hadrus (*formerly *Eubacterium hadrum)*	ATCC 29173^T^ (SS2/1)	80% (*n* = 27,000)	D-iLDH, butCoAT	Allen-Vercoe et al., 2012; Duncan et al., 2004
*Anaerostipes rhamnosivorans*	DSM 26241^T^	41% (*n* = 21,2000)	D-iLDH, butCoAT	Bui et al., 2014
*Coprococcus catus*	ATCC 27761^T^ (GD/7)	70% (*n* = 10,400)	Acrylate	Holdeman and Moore, 1974; Reichardt et al., 2014
*Eubacterium limosum*	DSM 20543^T^	56% (*n* = 16,400)	D-iLDH, butCoAT	Tanner et al., 1982
*Megasphaera elsdenii*	DSM 20460^Td^	42% (*n* = 7,290)	Acrylate	Elsden et al., 1956
*Veillonella parvula*	DSM 2008^Te^	52% (*n* = 46,000)	Succinate	Seeliger et al., 2002

a Alternative strain designations (indicated with =) and other strains given in brackets.

b Percentage of positive metagenome samples, total number of samples (*n*) in brackets (based on MAPseq; Rodrigues et al., 2017).

c Butyrate formation via NAD-independent D-lactate dehydrogenase (D-iLDH) and butyryl-CoA:acetate CoA-transferase (butCoAT), propionate formation via acrylate or succinate pathway (Figure 1).

d Rumen origin.

e Isolated from human mouth.

### Energetics of lactate metabolism

**Figure 2:**
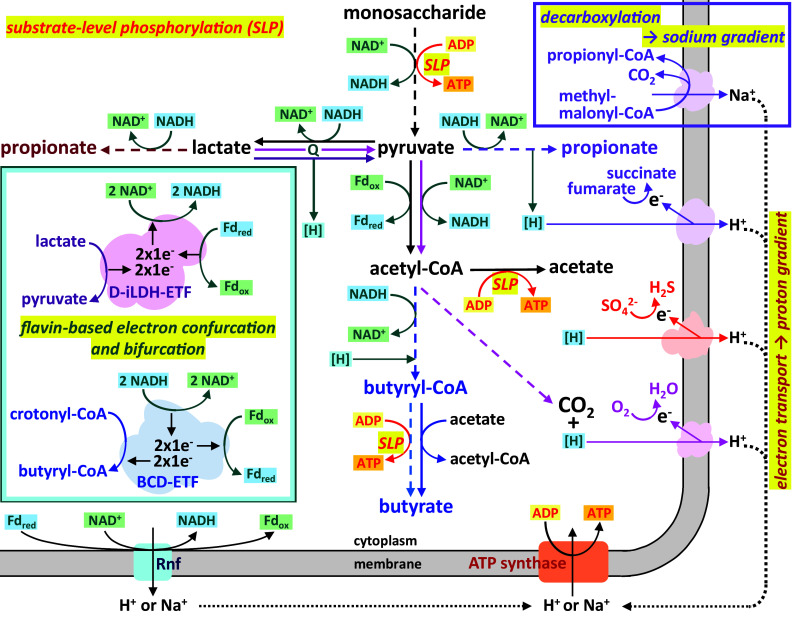
Principles of energy generation and flow of reducing equivalents during lactate metabolism. The general flow from substrates to key intermediates and products is shown rather than exact redox and ATP balances or stoichiometries. BCD, butyryl-CoA dehydrogenase; D-iLDH, NAD-independent lactate dehydrogenase; e^-^, electron; ETF, electron transfer flavoprotein; Fd_ox/red_, oxidised/reduced ferredoxin; [H], electrons/reducing equivalents without exact specification of electron carrier/coenzyme involved; NAD^+^/NADH, oxidised/reduced nicotinamide adenine dinucleotide; Q, quinone.

Organisms generate energy in the form of adenosine triphosphate (ATP) from the oxidation of growth substrates by several different mechanisms (Folch et al., 2021; Figure 2). Substrate-level phosphorylation by transfer of a phosphate residue from certain metabolic intermediates onto adenosine diphosphate takes place during some steps of glycolysis. It can also occur during the formation of acetate, propionate or butyrate from their respective CoA-esters, if this is carried out by two enzymes (a phosphotransacylase and an acyl kinase) via a phosphorylated intermediate. The alternative reaction of generating those acids by a CoA-transferase enzyme does not lead to direct formation of ATP, but as a new CoA-ester is formed during the reaction, the energy is essentially conserved. Thus, the butyryl-CoA:acetate CoA-transferase present in many intestinal butyrate producers results in the formation of butyrate and acetyl-CoA, and the acetyl-CoA may be further converted to acetate with concomitant ATP formation (Louis and Flint, 2017).

The reducing equivalents generated during the oxidation of substrates via glycolysis are transferred onto the electron acceptor NAD^+^, and the reduced NADH needs to be regenerated in order for glycolysis to continue operating. Aerobic microbes can make use of the reducing equivalents (and further ones generated by further oxidation of pyruvate to carbon dioxide) by channelling them through an electron transport chain situated in the cell membrane, which results in the reduction of the final electron acceptor oxygen to water and concomitant transport of protons across the membrane. The resulting proton gradient can then be used by ATP synthase (also resident in the membrane) to generate ATP or to fuel transport processes (Folch et al., 2021; Gottschalk, 1986; Figure 2). Certain anaerobes have electron transport systems that work with alternative final electron acceptors. Thus, sulphate-reducing bacteria utilise sulphate to generate sulphide (Tang et al., 2021), and propionate-producing bacteria following the succinate pathway link the step from fumarate to succinate to electron transport (Seeliger et al., 2002).

In the absence of electron transport chains, NAD^+^ can be regenerated by transfer of reducing equivalents from NADH onto metabolic intermediates, generating a range of fermentation products, including lactate, propionate and butyrate. Originally, it was thought that the sole reason for the generation of those fermentation products was the regeneration of the oxidised electron acceptor, but in recent years, enzyme systems have been discovered in many anaerobic microbes that enable further energy gain from some of these reactions (Buckel and Thauer, 2013). They enable the energetically unfavourable transfer of electrons between redox carriers of different redox potential (ie. the flow of electrons from NADH to ferredoxin) by coupling it to an exergonic reaction via a mechanism called flavin-based electron bifurcation (Figure 2). One of those enzyme systems operates during butyrate formation. The pathway intermediate crotonyl-CoA is reduced to butyryl-CoA with concomitant reduction of the electron carrier ferredoxin with reduced NADH, with the electron pair of each NADH molecule being bifurcated onto the two other co-reactants. The reduced ferredoxin can be re-oxidised by NAD^+^ reduction at the membrane complex Rnf under proton or sodium extrusion, resulting in an ion gradient that can be harnessed as described above (Buckel and Thauer, 2013). Several lactate-utilising bacteria make use of a lactate dehydrogenase enzyme complex that operates in the opposite direction, by electron confurcation, to enable the energetically unfavourable oxidation of lactate to pyruvate (Sheridan et al., 2022). The principle of electron bifurcation and confurcation is employed by various enzymes, including hydrogenases, and molecular hydrogen production and consumption also plays an important part in achieving redox balance and maximising energy gain. Thus, dissipating electrons as hydrogen allows for acetate production (accompanied by ATP generation by substrate-level phosphorylation) at the expense of more reduced fermentation products, but hydrogen can also serve as an electron donor (Folch et al., 2021).

Sodium ion extrusion also takes place during the decarboxylation of methylmalonyl-CoA to propionyl-CoA during propionate fermentation in *Veillonella* spp. (Seeliger et al., 2002; Figure 2). However, this bacterium requires the input of ATP for the first step in the pathway, the carboxylation of pyruvate to oxaloacetate, whereas other microbes employing this pathway utilise a transcarboxylation reaction linking both steps (Seeliger et al., 2002). The principles of energy generation are also not necessarily the same for analogous pathways. Thus, the acrylate pathway steps for propionate formation from lactate are analogous to the corresponding steps in butyrate formation, but while the reduction of crotonyl-CoA to butyryl-CoA involves electron bifurcation as detailed above, the analogous conversion of acryloyl-CoA to propionyl-CoA does not. This may have evolved to minimise the build-up of the highly reactive intermediate acryloyl-CoA (Buckel and Thauer, 2013). Furthermore, lactate conversion to pyruvate is carried out by different enzymes and redox co-factors in different bacteria, for example, a lactate dehydrogenase involving a membrane-bound menaquinone is employed by *Desulfovibrio* spp. (Tang et al., 2021).

### Lactate uptake

While lactate-utilising bacteria employ several different mechanisms to utilise lactate, they appear to all require a lactate permease to facilitate the cellular uptake of extracellular lactate. The gene encoding the lactate permease is therefore a promising marker gene for genomic prediction of lactate utilisation in microbes. Lactate permeases can be detected in a wide range of bacteria and archaea, and fall into four major phylogenetic protein families (with a fifth protein family containing permeases from eukaryotes; Sheridan et al., 2022). While there is a great diversity of lactate permeases across the tree of life, lactate permease family LP-IV is perhaps the most relevant in the human gut microbiota. This protein family is encoded by the prevalent commensal gut bacteria, *Anaerostipes*, *Anaerobutyricum*, *Megasphaera*, *Veillonella* and *Eubacterium limosum*, and also by the common gut pathogens *Campylobacter* and *Salmonella* (Table 2). However, when using lactate permease as a genetic marker gene of lactate utilisation, one must consider that the two proteins of the lactate permease superfamily encoded by *Escherichia coli* K12 are also involved in the uptake of glycolate (Núñez et al., 2002). This raises the possibility that lactate permeases may be present in some organisms that utilise glycolate but lack the capacity to utilise lactate.

**Table 2.** Bacterial genera found in the mammalian gut containing strains that possess at least one family LP-IV lactate permease gene, based on analysis of available genomes (Sheridan et al., 2022).

Phylum	Genus	Number of species^a^	Copies in genome
Bacteroidetes	*Bacteroides*	4	1
Bacteroidetes	*Prevotella*	3	1
Firmicutes	*Anaerobutyricum*	2	1
Firmicutes	*Anaerostipes*	4	1
Firmicutes	*Bacillus*	25	1 or 2
Firmicutes	*Clostridium*	12	1
Firmicutes	*Coprococcus*	2	1 or 4
Firmicutes	*Dorea*	1	1
Firmicutes	*Eubacterium*	3	1
Firmicutes	*Intestinibacter*	1	1
Firmicutes	*Lactobacillus*	15	1
Firmicutes	*Megasphaera*	6	1 or 2
Firmicutes	*Mitsuokella*	1	1
Firmicutes	*Selenomonas*	3	1 or 2
Firmicutes	*Veillonella*	2	1
Proteobacteria	*Campylobacter*	7	1
Proteobacteria	*Citrobacter*	1	1
Proteobacteria	*Desulfovibrio*	9	1
Proteobacteria	*Escherichia*	1	2
Proteobacteria	*Helicobacter*	7	1
Proteobacteria	*Salmonella*	1	1
Proteobacteria	*Vibrio*	1	1

a Number of species found to carry a predicted lactate permease (Sheridan et al., 2022).

## Lactate metabolism in intestinal microbial communities

As we have seen, lactate produced by microbial fermentation or host metabolism can be exploited by a variety of bacterial groups within the gut microbiota as a carbon or energy source, or as a co-substrate. This must create competition for lactate between different lactate utilisers. For example, lactate is a favoured co-substrate for sulphate-reducing bacteria such as *Desulfovibrio* spp., which have the capacity for anaerobic respiration. In the presence of sulphate, this leads to the formation of sulphide that can be toxic for the colonic epithelium. Marquet et al. (2009) explored competition for lactate in a triculture between a lactate producer (*Bifidobacterium adolescentis*), *Desulfovibrio piger*, and a butyrate-forming lactate utiliser, namely *Anaerobutyricum soehngenii* [formerly *Eubacterium hallii* and reclassified by Shetty et al. (2018)]. Butyrate production from lactate was partially inhibited by *D. piger*, but sulphide formation was unaffected by the presence of *A. soehngenii.* Lactate metabolism by the complex microbiota is also affected by the prevailing gut pH (Belenguer et al., 2007), which will be covered below.

The existence of lactate racemases in many lactate-utilising bacteria might permit the interconversion of the two stereoisomers, L- and D-lactate, which could be important given the stereospecificity of many of the enzymes involved in lactate utilisation (Sheridan et al., 2022). Nevertheless, some lactate-utilising bacteria, for example, *Anaerostipes hadrus,* lack lactate racemase activity and utilise only D-lactate (Duncan et al., 2004). An intriguing possibility is that there might be special associations between particular producers and utilisers of D- or L-lactate, but this remains unknown.

### Regulation of lactate utilisation

For most, if not all, lactate-utilising bacteria, lactate is just one of many alternative substrates, and substrate preferences may be influenced by availability of these various energy sources *in vivo.* In *A. soehngenii*, expression of the *lct* gene cluster responsible for lactate utilisation (Shetty et al., 2020) is strongly induced during growth on lactate as the only added energy source, but repressed when excess glucose is supplied along with lactate (Sheridan et al., 2022). In the large intestine, however, glucose concentrations are considered to be generally very low because of its efficient uptake by the host in combination with rapid utilisation by the microbial community. This concentration dependence of repression of lactate utilisation by glucose has not been investigated in detail, but it seems likely that low concentrations of glucose or other carbohydrates will not prevent lactate being used altogether. An indication of this comes from the observation that in co-cultures between *A. soehngenii* (formerly *E. hallii*) and *B. adolescentis* growing on starch, lactate produced by *B. adolescentis* was converted completely into butyrate (Duncan et al., 2004). Less repression of D-lactate utilisation was seen by glucose in pure culture of *A. hadrus* (Muñoz-Tamayo et al., 2011).

The *lct* gene cluster of *A. soehngenii* encodes lactase permease, an NAD-independent lactate dehydrogenase (D-iLDH), two electron transfer flavoproteins (ETFs), an orthologue of butyryl-CoA dehydrogenase (BCD) and lactate racemase, which all show co-ordinated upregulation during growth on lactate (Shetty et al., 2020). At the same time, expression of the BCD orthologue encoded by the central butyrate pathway cluster, together with its two adjacent ETF proteins, was found to be downregulated on lactate (Sheridan et al., 2022). This suggests that the *lct*-encoded BCD may largely replace the function of the “normal” orthologue when lactate replaces sugars as the energy source. It seems likely that this must somehow help to link electron transfer between the reactions catalysed by BCD and the co-ordinately upregulated D-iLDH.

The six genes of the newly identified *lap* gene cluster responsible for lactate conversion to propionate via the acrylate pathway in *C. catus* were also highly induced by growth on lactate (Sheridan et al., 2022). These genes are assumed to encode for propionyl-CoA transferase, lactoyl-CoA epimerase, lactoyl-CoA dehydratase and lactate permease (Sheridan et al., 2022). The *C. catus* GD/7 genome also contains a truncated set of *lct* genes that showed a lower amplitude of upregulation by lactate. Repression of lactate utilisation by hexose sugar (fructose) was less than that observed in *A. soehngenii* (on glucose).

### Ecosystem modelling of interplay between lactate metabolism and pH

Theoretical modelling has been used to simulate the utilisation of lactate and acetate by pure cultures of butyrate-producing lactate utilisers (Muñoz-Tamayo et al., 2011). More ambitiously, a theoretical model of the whole human colonic microbial community has been developed by representing it as ten microbial functional groups (MFGs), defined by their fermentative metabolism. These MFGs can be correlated approximately with phylogenetic groups defined from sequence data. This model was first used to simulate the impact of a one-unit pH shift on community composition and metabolism observed experimentally in *in vitro* continuous culture (Kettle et al., 2015; Walker et al., 2005). Two MFGs were assumed to utilise lactate, producing propionate and butyrate, respectively, but the model has since been refined to include selective growth inhibition of different MFGs by lactate. Ki values for MFGs that do not produce lactate were derived from experimental evidence in pure cultures, with the Bacteroidetes group showing the greatest growth inhibition by lactate, especially at slightly acidic pH (Wang et al., 2020).

These computer simulations helped to explain two key findings from experiments involving human colonic microbial communities maintained in pH-controlled continuous flow fermentors (Wang et al., 2020). First, at mildly acidic pH (5.5), the system tended to become chaotic, with lactate producers increasing and lactate accumulating, while other anaerobic bacteria that produce propionate and butyrate decreased dramatically in their relative abundance. This behaviour represents lactate-induced perturbation, similar to the situation that occurs in ruminants suffering from lactic acidosis. Second, the abundance and activity of the two lactate utiliser groups was predicted to be critical to the stability of the community, ie. in preventing, or reversing, such perturbations. Interestingly, we found experimentally that restoration of the initial community balance could occur during constant infusion of 10-mM DL lactate, presumably through the impact of these lactate-utilising groups in reversing lactate accumulation. At more neutral pH (6.5), the presence of sufficient lactate utiliser populations prevented the accumulation of lactate even with continuous infusion of 20-mM lactate (Wang et al., 2020). Taken together, these findings provide further evidence that lactate-utilising bacteria can play a key role in maintaining colonic health by preventing accumulation of lactate and therefore subsequent microbial community perturbations.

## Health/disease implications

Lactate is usually detected as a minor metabolite in the healthy human colon (<4 mM; Hove and Mortensen, 1995). Of the microbes producing lactate as a major fermentation product, species belonging to the Lactobacillales, predominantly streptococci and lactobacilli, are prevalent in the small intestine, but proportionally less abundant in the large intestine (Heeney et al., 2018). Importantly, bifidobacteria are usually more prevalent in the human large intestine, particularly in breast-fed infants where they are the dominant genus, although it should be noted that they are commonly detected at less than 5–10 per cent of the microbiota in adults (Arboleya et al., 2016). Wang et al. (2014) reported that there was an increase in the abundance of bifidobacteria and lactobacilli in active inflammatory bowel disease (IBD) with a concomitant decrease in the abundance of butyrate-producing species and an increase in faecal lactate levels. As detailed above, other intestinal bacterial species can, however, cross-feed on lactate, with levels of lactate in the large intestine reflecting the balance between lactate produced and lactate utilised. Lactate can exert both positive and negative effects on the gut environment (summarised in Table 3), and maintaining the right balance between production and utilisation therefore appears important for disease prevention.

**Table 3.** Summary of possible consequences of microbial lactate production in the gut.

Positive	Reference(s)
Stimulation of butyrate and propionate formation via lactate cross-feeding by other members of the gut microbiota	Duncan et al., 2004; Reichardt et al., 2014; Sheridan et al., 2022
Decreased gas production from fermentation of carbohydrates	Kamke et al., 2016
Inhibition of pathogens such as *E. coli* O157	McWilliam Leitch and Stewart, 2002
Modulation of host immunity and gut epithelial development	Garrote et al., 2015; Lee et al., 2018
Negative	Reference(s)
Neurotoxicity of D-lactate	Chan et al., 1994
Destabilising impact on the gut microbiota through low pKa and differential growth inhibition; can lead to lactic acidosis, which is potentially fatal	Duncan et al., 2009; Nocek, 1997; Wang et al., 2020
Growth substrate for some pathogenic bacteria, e.g. *Salmonella*, *C. jejuni*	Gillis et al., 2019; Thomas et al., 2011
Co-substrate for sulphate-reducing bacteria, resulting in enhanced production of genotoxic H_2_S	Marquet et al., 2009; Tang et al., 2021
Possible promotion of virulence in some pathogens (e.g. *C. albicans*)	Ballou et al., 2016; Llibre et al., 2021

### Lactate accumulation, health and disease in the mammalian gastrointestinal tract

In ruminant animals (eg. cattle and sheep), lactate accumulates when large amounts of readily fermentable substrates, such as starch and sugars, enter the rumen. Rapid fermentation by lactate-producing bacteria, such as *Streptococcus bovis*, can drive down the rumen pH as the dissociation constant for lactate is pKa 3.85, which is lower than that of the other major fermentation acids detected in mammalian stool samples. As outlined above, such reductions in prevailing pH have the potential to significantly perturb the resident microbiota as, when pH values drop below 6, this is likely to inhibit the growth of most of the microbes that dominate the rumen microbial community, including key groups of lactate-utilising bacteria (Wang et al., 2020). This loss of lactate-utilising potential further exacerbates lactate accumulation, which in turn creates more favourable conditions for the proliferation of the lactate-producing bacteria that are able to tolerate the lower pH (Russell and Rychlik, 2001). Moreover, some of the lactate-producing bacteria form the D-form of lactate, which is a neurotoxin and also contributes to the acidosis in the animal. Studies in cattle (Shabat et al., 2016) and sheep (Kamke et al., 2016) indicate a link between lactate production and utilisation, feed conversion efficiency and methane production. They suggest a combined shift towards lactate-producing and lactate-utilising bacterial activity (mainly associated with *M. elsdenii* and *C. catus*) within the rumen microbiota in higher productivity animals that show lower methane production. The explanation for this is thought to be that carbohydrate fermentation involving lactate cross-feeding yields less hydrogen and therefore less methane as compared with fermentation by hydrogen-producing microorganisms. The carbon is routed instead mainly towards propionate, which helps to increase the supply of energy from the diet to the host.

In hindgut fermenters, such as horses, rapid dietary changes in feed patterns, such as fructan-rich pasture, can also disrupt normal fermentation in the hind gut causing lactic acidosis and colic. This can also predispose animals to laminitis, a painful condition resulting from inflammation of the tissue inside the hoof of animals. It accounts for around 15 per cent of all lameness in horses and can be fatal (Nocek, 1997). As in ruminants, lactate levels in the hindgut are normally low due to the activity of lactate-utilising bacteria, but lactate accumulation and a pH drop potentially allow pathogens to proliferate. Bacteria belonging to the *Veillonellaceae* family, including *M. elsdenii*, may play a role in utilising lactate in horses and have been considered for development as a treatment for lactic acidosis in these animals (Douthit et al., 2019).

In humans, babies that are breast fed are colonised by a high proportion of bifidobacteria, which will form lactate as a fermentation end product. The importance of gut bacteria to metabolise lactate in the infant intestinal tract is likely to be high as lactate accumulation has been associated with colic in infants (Pham et al., 2019). Furthermore, the lactate utiliser *A. caccae* may also have a protective role against the development of food allergy (Feehley et al., 2019). In all age groups, lactate accumulation is associated with surgical removal of portions of the small and large intestine in humans (Kowlgi and Chhabra, 2015), and with gut disorders such as the IBDs, Crohn’s disease and ulcerative colitis (Hove et al., 1994). There is also evidence to suggest that lactate accumulation correlates with disease severity in IBD. Vernia et al. (1988), for example, showed that ulcerative colitis patients with severe symptoms had elevated faecal lactate concentrations, in tandem with reduced concentrations of short-chain fatty acids, such as butyrate, compared to patients with mild or quiescent disease. Lactate may also be produced by host tissues, although the relative contributions of bacterial and host sources are at present unclear. Studies in germ-free mice revealed lactate in caecal samples of mice, indicating that lactate can come from the host tissues, but the levels measured were low (0.38 mM; Gillis et al., 2018). This suggests that much of the lactate detected in patients with IBD is formed by intestinal bacteria.

### Role of lactate in colonisation resistance against gastrointestinal pathogens

The gut microbiota acts as a potent barrier against colonisation and/or proliferation of gastrointestinal pathogens via a multifactorial process called “colonisation resistance.” Colonisation resistance can be mediated by direct microbiota–pathogen interactions, for example, the release of antimicrobial compounds that are active against invading pathogens, via competition for carbon sources, micronutrients and binding sites, or indirectly via microbiota-induced stimulation of the host immune system and/or gut barrier (Lawley and Walker, 2013).

Lactate may play multiple important roles in colonisation resistance. On the one hand, it may have beneficial effects, as lactate has been shown to inhibit the growth of many foodborne pathogens (Vieco-Saiz et al., 2019), and reduction in colonic pH caused by production of lactate (and other fermentation acids) by predominant bifidobacteria is considered to be a potentially protective factor in breast-fed infants (Sarkar and Mandal, 2016). In contrast, the pH-lowering activities and potential toxicity of lactate also have the potential to perturb the wider gut microbiota, and thereby reduce the potency of colonisation resistance. As described above, recent work using pH-controlled continuous culture fermentor systems has shown the dramatic impact that elevated concentrations of lactate in combination with mildly acidic pH can have on the colonic microbiota, resulting in depletion of many key obligately anaerobic bacterial lineages, and of health-associated short-chain fatty acids such as butyrate and propionate (Wang et al., 2020).

Importantly, a number of bacterial gastrointestinal pathogens of humans and animals, such as *Salmonella* spp., *E. coli*, *Campylobacter jejuni, Clostridioides difficile* and *Vibrio cholerae*, appear to be able to utilise lactate for growth (Gillis et al., 2019; Thomas et al., 2011; Table 2). This may give these pathogens a competitive advantage in situations where gut lactate is elevated, such as in IBD (Vernia et al., 1988) or following antibiotic treatment or diarrhoeal disease (Gillis et al., 2018), particularly when the indigenous microbiota is depleted and colonisation resistance is temporarily reduced. Proof of concept work in mouse models has demonstrated that depletion of butyrate-producing members of the gut microbiota can result in elevated release of L-lactate and oxygen by gut epithelial cells into the lumen, providing a more favourable environment for growth of the pathogen *Salmonella* Typhimurium (Gillis et al., 2018). Similarly, cholera toxin may promote *Vibrio cholerae* in the gut via, among other mechanisms, increased release of lactate by perturbed host cells, which may then be utilised for growth by the pathogen (Rivera-Chávez and Mekalanos, 2019).

In addition to impacting several important bacterial pathogens, lactate may also increase the virulence of the fungal pathogen *Candida albicans*, either by acting as a carbon source for growth, or by enhancing biofilm formation and resistance to antifungal compounds (Llibre et al., 2021). Exposure to lactate also triggers masking of key cell wall antigens by *C. albicans*, thereby making the pathogen less visible to the immune system (Ballou et al., 2016). Thus, lactate appears to have multiple potential impacts on colonisation resistance, either by inhibiting growth of the beneficial indigenous microbiota, or by enhancing growth and/or altering gene expression to enhance virulence of numerous pathogens.

### Lactate-utilising bacteria as a source of new probiotics

As outlined above, with the exception of breast-fed infants, lactate accumulation in the gut is generally associated with a range of detrimental health outcomes. Depletion of key lactate-utilising bacteria is also associated with poor health. Recently, Wirbel et al. (2021) developed a machine learning tool for meta-analysis of metagenomic studies comprised of more than 10,000 human faecal samples, which revealed that lactate-utilising *Anaerostipes* species are frequently depleted across multiple diseases, including gastrointestinal diseases. These lactate-utilising species may therefore offer promise as candidates for the development of next-generation probiotics (Gilijamse et al., 2020; Wang et al., 2020), particularly if they can be administered prior to accumulation of lactate and widespread perturbation of the gut ecosystem. Furthermore, modelling studies with marathon runners illustrate that systemic lactate produced during exercise crosses the gut wall and is metabolised by *Veillonella* species to form propionate, and it has therefore been suggested that this lactate-utilising bacterium may help to promote athlete performance (Scheiman et al., 2019).

At present though, very few trials have been performed to demonstrate the efficacy of lactate-utilising bacteria as probiotics in human subjects. However, Gilijamse et al. (2020) recently conducted a small-scale human study that showed that orally dosed *A. soehngenii* improved peripheral insulin sensitivity, which was accompanied by altered microbiota composition. Importantly, the probiotic could be detected in stool samples provided by the recipients, suggesting survival through the gastrointestinal tract, and was deemed safe. Duodenal infusion of *A. soehngenii* in individuals with metabolic syndrome revealed that it stimulated the insulinotropic hormone glucagon-like peptide 1 production and increased expression of regenerating islet-protein 1B (Koopen et al., 2021).

In addition to efficacy testing, there are other important barriers to overcome before obligately anaerobic lactate-utilising bacteria could be widely adopted as novel probiotics. Most species are highly oxygen sensitive, meaning novel delivery methods may need to be developed. In addition, unlike traditional probiotics, such as lactobacilli and bifidobacteria, which are generally regarded as safe, comparatively much less is known about most anaerobic lactate-utilising bacteria, and extensive safety testing and screening for undesirable traits such as antibiotic resistance genes and virulence factors will likely be required. Nonetheless, many species, particularly those that can convert lactate into the beneficial short-chain fatty acid butyrate, are undoubtedly appealing candidates for further development (Van Immerseel et al., 2010).

Another approach that is attracting interest to treat certain disease conditions, particularly bowel disorders, is faecal microbiota transplantation (FMT), which is the transfer of faecal bacteria from a healthy donor to another individual. For example, Paramsothy et al. (2019) reported that FMT in ulcerative colitis patients achieving remission resulted in enrichment of several species including two lactate-utilising bacteria, namely *A. hallii* and *A. hadrus*, when compared to patients that did not achieve remission.

## Conclusions

The acidic products of anaerobic fermentation are a critical factor in the gut environment, affecting both the host and the microbial community that produces them. Lactate production in the gut has both possible benefits and potentially negative consequences as elaborated above (and summarised in Table 3). Furthermore, the low pKa and selective microbial growth inhibition of lactate can precipitate dramatic shifts in the species composition and metabolic outputs of gut microbial communities. In the adult human colon, a balance between lactate production and utilisation that avoids lactate accumulation therefore appears to be important for health and the activities of specialised lactate-utilising bacteria, discussed here, help to maintain stability by preventing lactate accumulation. Differences in lactate-utilising bacterial populations between individuals may therefore have important consequences for the stability of their gut microbial communities. In addition, lactate-utilising species create cross-feeding pathways that can influence system responses to dietary changes. The fact that lactate can be routed by lactate-utilising bacteria into butyrate and propionate means that the promotion of lactate-producing bacteria may indirectly promote the formation of these two acids, potentially delivering benefits for health and nutrition. Metabolism of carbohydrates by this route may also result in lower net formation of hydrogen, CO_2_ and methane, a consideration of particular significance in relation to ruminant nutrition. The fact that lactate is also an energy source for many pathogens and a co-substrate for sulphate-reducing bacteria, however, adds further complexity, and more attention needs to be paid to understanding the competition for lactate between commensal and pathogenic organisms within the gut community.

## Data Availability

Data sharing is not applicable to this article as no new data were created or analysed.
